# Monoallelic characteristic-bearing heterozygous L1053X in *BRCA2* gene among Sudanese women with breast cancer

**DOI:** 10.1186/s12881-017-0448-x

**Published:** 2017-08-16

**Authors:** Alsmawal A. Elimam, Mohamed Elmogtba Mouaweia Mohamed Aabdein, Mohamed El-Fatih Moly Eldeen, Hisham N. Altayb, Mohamed Adel Taha, Mohammed N. Nimir, Mohamed D. Dafaalla, Musaab M. Alfaki, Mohamed A. Abdelrahim, Abdelmohaymin A. Abdalla, Musab I. Mohammed, Mona Ellaithi, Muzamil Mahdi Abdel Hamid, Mohamed Ahmed Salih Hassan

**Affiliations:** 1 Post-graduate College, Al-Neelain University, Khartoum, Sudan; 2 Daoud Research Group, Khartoum, Sudan; 3 Department of Histopathology and Cytology, Faculty of Medical Laboratory Sciences, Alneelain University, Khartoum, Sudan; 4 Faculty of medical laboratory, Sudan University of science and technology, Khartoum, Sudan; 5 Department of Bioinformatics, Africa City of Technology, Sudan University of Medical Science and Technology, Khartoum, Sudan; 6 Institute of Endemic Diseases, University of Khartoum, Khartoum, Sudan; 7 Department of Internal Medicine, Faculty of Medicine, University of Khartoum, Khartoum, Sudan; 8 Ahfad University for Women, Omdurman, Sudan

**Keywords:** *BRCA2*, Monoallelic, Heterozygous, Stop Codon, Breast cancer, Sudanese patients

## Abstract

**Background:**

Breast cancer (BC) is the most common type of cancer in women. Among many risk factors of BC, mutations in *BRCA2* gene were found to be the primary cause in 5–10% of cases. The majority of deleterious mutations are frameshift or nonsense mutations. Most of the reported *BRCA2* mutations are protein truncating mutations.

**Methods:**

The study aimed to describe the pattern of mutations including single nucleotide polymorphisms (SNPs) and variants of the *BRCA2* (exon11) gene among Sudanese women patients diagnosed with BC. In this study a specific region of *BRCA2* exon 11 was targeted using PCR and DNA sequencing.

**Results:**

Early onset cases 25/45 (55.6%) were premenopausal women with a mean age of 36.6 years. Multiparity was more frequent within the study amounting to 30 cases (66.6%), with a mean parity of 4.1. Ductal type tumor was the predominant type detected in 22 cases (48.8%) among the reported histotypes. A heterozygous monoallelic nonsense mutation at nucleotide 3385 was found in four patients out of 9, where TTA codon was converted into the stop codon TGA.

**Conclusion:**

This study detected a monoallelic nonsense mutation in four Sudanese female patients diagnosed with early onset BC from different families. Further work is needed to demonstrate its usefulness in screening of BC.

## Background

Breast cancer (BC) is the most commonly diagnosed type of cancer in women, accounting for 25% of all cancer cases in the world; with much more cases recorded in developing countries than developed ones. In 2012, 1.67 million cases of BC resulted in 522,000 deaths [1–3]. In Africa, 324,000 deaths were reported to be caused by BC [1, 2]. The predisposition to BC appears to be affected by several factors, one of them is the high-risk BC gene mutation in *BRCA2* (OMIM: 600185) (Gene ID: 675) (RefSeqGene: NG_012772) [4]. Although the incidence rate of gene mutation in *BRCA2* is low, it is associated with a high lifetime risk of BC [5, 6]. This lifetime risk is variable among different population [7–10]. *BRCA2* is believed to be the primary cause of 5 to 10% of all cases of BC [11]. About 45% of women, who inherited a defective *BRCA2* allele, will develop BC when they reach the age of 70 [12, 13]. Mean age at onset of BC for *BRCA2* mutation carriers is reported to be 42.8 years [14].

The human *BRCA2* gene contains 27 exons, among which exon 11 is the largest one. The coding sequence (RefSeq transcript mRNA: NM_000059) was determined to be 11,385 bp, which codes for a protein of 3418 amino acids (Uniprot: P51587) (RefSeq protein: NP_000050) [15]. A study conducted in Central Sudan from 2001 to 2002 concluded that this gene plays a role in the etiology of BC [16]. In addition, in a genetic analysis performed on secondary school female students in Northern Sudan, some variants were detected in two groups free of BC, one with a family history of BC and the other without familial risks. Two *BRCA2* mutations were reported in the group without a family history [17].

It is known that the majority of deleterious mutations in *BRCA2* are either a frameshift or nonsense mutations [14, 18, 19]. The nonsense mutations have been reported more within exon 11 of early onset BC cases with high pathogenicity [14, 18]. It is found that about 90% of reported *BRCA2* mutations are protein truncating [20]. In addition, the formation of nonsense-mediated RNA decay -as premature terminating inactivation codon- could lead to the production of a toxic partial protein [14]

Heterozygosity of *BRCA2* mutations was found to be associated with a distinctive phenotype, which could lead to *BRCA2* tumorigenesis, as altered heterozygous *BRCA2* does not function well and the wild allele alone is not enough to maintain genomic stability. In other cases, it was suggested to be haploinsufficient. Furthermore, *BRCA2* monoallelic carrier mutations were detected in patients with pancreas and breast cancer [21, 22].

Etiologically, scientific literature from African countries showed that reproductive factors more commonly associated with the development of BC are early menarche, pregnancy, and multiparity [23]. The situation is globally similar; as early menarche, late menopause, carriers of *BRCA2* damaging variants, and early pregnancy before age of 30 years confer high-risk conditions for BC [24].

Unfortunately, the scientific articles from African countries lacked data about the risk conferred by familial cases as it has not been well investigated, although some studies suggested its etiological companion [16, 23]. This study aimed to screen *BRCA2* mutations, taking into consideration the biggest region in the gene, exon 11, to find out and investigate variants or single nucleotide polymorphisms (SNPs) among known BC patients.

## Methods

### Study area

This study was carried out in Khartoum state at the Radiation and Isotope Center in Khartoum (RICK), which is one of the only two oncology centers in Sudan, and it provides oncological services for people from all parts of Sudan.

### Sampling

Out of all Sudanese female patients diagnosed with BC (45 patients) attending RICK during March 2015, 10 patients were selected randomly for genetic sequencing and analysis. Four healthy subjects with no family history of BC and another one diagnosed with essential thrombocythemia who are free of BC have been added as controls. Blood specimens were collected using EDTA-vacutainer tubes from the selected patients and controls. The specimens were preserved at −20 °C.

### Ethical considerations

All patients were informed and consented to participate in the study before collecting the samples. All patients were consented to publish the results of the study. Ethical approval was obtained from the ethical committee of Sudan Ministry of Health-Khartoum state.

### DNA extraction

For both patients and controls, DNA was extracted by Salting out technique according to the published protocol [25]. In addition, we added proteinase K at 56 °C to enhance white cells membrane breakdown. After 1 h, the DNA was extracted with concentration of 30 ng/ul, dissolved in 100 ul Tris-EDTA (TE) Buffer, and kept for overnight at 4 °C, then preserved at −20 °C until use.

### PCR amplification

**Fig. 1:**
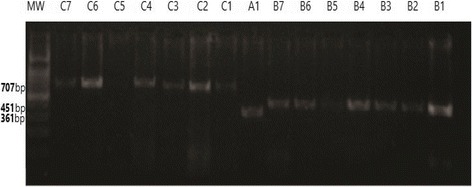
Illustrates PCR amplification results of the three tested regions (**A**, **B** & **C**) on 2% gel electrophoresis. MW: DNA ladder’s molecular weight where 100 bp was used. C7 to C1 lanes indicate primer C bands. A1 indicates primer A band. B7 down to B1 Lanes indicate primer B bands

Forty-five patients and five control samples were subjected to amplification using three primers sets (A, B and C) targeting three regions within *BRCA2* gene exon 11 as described in (Table 1). This study focused only on the product of the second primer set (primer B) based upon stability and quality of this primer [26]. Primers were synthesized and purchased from Macrogen Incorporation (Seoul, South Korea). Annealing temperature was adjusted using Maxime PCR PreMix Kit i-Taq 20 μl (INTRON Biotechnology, South Korea) on several runs of PCR. The adjusted temperatures are described in (Table 1). Amplification for the targeted regions was done after addition of 15 ul Distilled water, 3 ul sample DNA and 1 ul of each forward and reverse to the ready-to-use master mix volume. PCR mixture was subjected to an initial denaturation step at 96 °C for 5 min, followed by 35 cycles of denaturation at 96 °C for 30 s, primer annealing at 50 °C for 30 s, followed by a step of elongation at 72 °C for 60 s, the final elongation was at 72 °C for 10 min [26]. The PCR products were checked and analyzed by 2% agarose gel electrophoresis at 100 V for 30 to 45 min and then bands were visualized by automated gel photo documentation system (Fig. 1). Only 10 patients and five controls yielded sufficient quality bands, and were subsequently selected for sequencing by the Sanger sequencing technique.

**Table 1:** List of primers used to amplify *BRCA2* gene selected regions

PN*	Primer’s nucleotide sequence	PL*	AT*	Selected region	Am*
A	F: 5′ AGA CAC AGG TGA TAA ACA AG ‘3	20	50	3020 to 3380	361
	R: 5′ CAA GGT ATT TAC AAT TTC AA ‘3	20	50		
B	F:5′ GCT CTC TGA ACA TAA CAT TAA G ‘3	22	50	3281 to 3731	451
	R: 5′ CAT TAT GAC ATG AAG ATC AG ‘3	20	50		
C	F: 5′ TGA GAC CAT TGA GAT CAC AGC ‘3	21	55	4967 to 5673	707
	R: 5′ TAG TCA CAA GTT CCT CAA CGC A ‘3	22	55		

PN* Primer Name

PL* Primer length in base pair

AT* Annealing Temp

Am* Amplicon size (bp)

### Sequencing of *BRCA2* gene

Sanger sequencing was performed for the PCR products. Both DNA strands were sequenced by Macrogen Company (Seoul, South Korea).

### Bioinformatics analysis

For each sample, the two purified chromatogram (forward and reverse) nucleotide sequences were viewed and checked for quality by FinchTV program version 1.4.0 [27]. The NCBI Nucleotide database was searched for reference sequences. *BRCA2* nucleotide sequence (NM_000059.3) was obtained and all regions were analyzed accordingly [10]. Additional high similarity sequences (AY436640.1) and (X95161.1) were obtained from NCBI database and were added as control sequences using nucleotide Basic Local Alignment Search Tool (BLAST) [28]. Any apparent changes within the tested sequences were noticed through multiple sequence alignment using BioEdit software [29]. All sequences were translated into amino acid sequences using online Expasy translate tool [30]. The resulted amino acid sequences were compared all together using BioEdit software.

### SNP prediction

SIFT-software was used to check for the effect of SNPs on the protein; whether they are damaging or not [31]. Also, SNPs structural and functional impact on resultant protein was predicted by PolyPhen-2; which performs searches in several protein structure databases for 3D protein structures, multiple alignments of homologous sequences and amino acid contact information. [32]

Project hope was used to analyze the structural and conformational variations that have resulted from single amino acid substitutions corresponding to the single nucleotide substitutions [33], then the protein stability was assessed by I-Mutant [34], In addition to web-based applications for rapid evaluation of the disease-causing potential of DNA sequence alterations called MutationTaster2 [35]

## Results

### Study population characteristics

#### Patient characteristics, clinical and histological parameters

Forty-five women with BC, who attended RICK-center for treatment and follow-up, were selected for the study, their age ranged between 27 to 80 years (mean age was 45.9 years). Out of 45 patients, 25 (55.6%) were premenopausal women (Early onset cases) with a mean age of 36.6 years. On the other hand, late onset cases - who were 46 years or more - had a mean age of 57.4 years. The majority of women in the study were multiparous 30/45 (66.6%), with an average number of 4.1 parities. Patients were from 17 tribes, Ja’alya, Shaygeya, and Dnagla were the most frequent tribes (Table 2). Familial history of any type of cancer was found in 11 cases; of which six cases had BC in the family. Abortion was detected in 10 cases (22.2%), with an estimated frequency of 1–5 times. Among the married cases (88.8%), three cases were married at less than 20 years of age.

**Table 2:** Patients demographic and characteristics

Variable		Frequency (%)
Onset	Early (≤45 years)	25/45 (55.6%)
	Late (≥46 years)	20/45 (44.4%)
Family history	Breast cancer	6/45 (13.3%)
	Other cancer	5/45 (11.1%)
	No family history of any cancer	34/45 (75.6%)
Parturition	Multiparous	30/45 (66.7%)
	Nulliparous	13/45 (28.9%)
	Primiparous	2/45 (4.4%)
History of Abortion	Yes	10/45 (22.2%)
	No	35/45 (77.8%)
Marital status	Currently Married	41/45 (91.1%)
	Single	3/45 (6.7%)
	Previously married	1/45 (2.2%)
Tribe	Ja’alya	5 (11.1%)
	Shaygeya	5 (11.1%)
	Dnagla	4 (8.9%)
	Noba	3 (6.7%)
	Rezaigat	3 (6.7%)
	Others	25 (55.5%)
Geographical region	Central Sudan^a^	21/45 (46.7%)
	Western Sudan	15/45 (33.3%)
	Northern Sudan	6/45 (13.3%)
	Eastern Sudan	3/45 (6.7%)
Tumor site	Unilateral	35/45 (77.8%)
	Bilateral	4/45 (8.9%)
	Unknown	6/45 (13.3%)

^a^Comprising both Khartoum 16 cases and AlGezirah 5 cases

Available histotype data showed that ductal tumors were the predominant type (detected in 22 cases (48.8%)). Lobular and mucinous were reported in 5 and 2 cases respectively. Papillary adenocarcinoma was detected in only one patient, as a secondary deposit in bone. The right side was affected by the disease in 20 patients (44.4%). Four patients had bilateral disease (Table 2).

Mean age at diagnosis in the group selected for DNA sequencing was 39 years (27 to 57 years). Nine patients were multiparous (mean of parity was 3.5). In this group, while the right-side was predominantly affected, one patient had bilateral breast involvement. Cancer grades were between II to III. Clinical staging showed lymph nodes involvement in five cases. Distal metastasis was noted in the liver in one patient; while bone and lung involvement were documented in another case. Control individuals were free of BC and free of family history involvement. The youngest patient within the study was 27 years old and was the only case free of lymphatic involvement (Table 3).

**Table 3:** The highly purified Breast Cancer Patients demographic, clinical, histological parameters with the nonsense mutation

Patients ID	Age	Family history of BC	grade	Stage and Metastasis	histotype	BC site	Nonsense Mutation
B1	51	second and third degree^*1^	NA	T_1_N_1_M_0_	NA	Rt/Unilateral	
B2	45	No	NA	T_x_N_x_M_x_	NA	Bilateral	Detected
B13^*2^	27	No	III	T_2_N_1_M_0_	Ductal	Lt/Unilateral	
B14	35	Second degree	NA	T_x_N_x_M_1_ (Liver)	Lobular	Rt/Unilateral	Detected
B18	41	No	II	T_x_N_x_M_x_	Ductal	Rt/Unilateral	
B23	27	No	NA	T_2_N_0_M_0_	NA	Rt/Unilateral	Detected
B24	39	No	NA	T_x_N_x_M_x_	NA	Rt/Unilateral	Detected
B29	37	No	III	T_x_N_x_M_x_	Ductal	Rt/Unilateral	
B39	30	No	II	T_4_N_1_M_x_	Ductal	Lt/Unilateral	
B44	57	No	I	T_x_N_x_M_1_ (Bone/Lung)	Ductal	Rt/Unilateral	

^*1^Two of the relatives involved by breast cancer

^*2^This patient was excluded from bioinformatics analysis due to inconsistency and poor quality

### Bioinformatics result analysis

The sequencing data was checked for consistency and quality, and one patient’s sequence has been excluded for inconsistency.

**Fig. 2:**
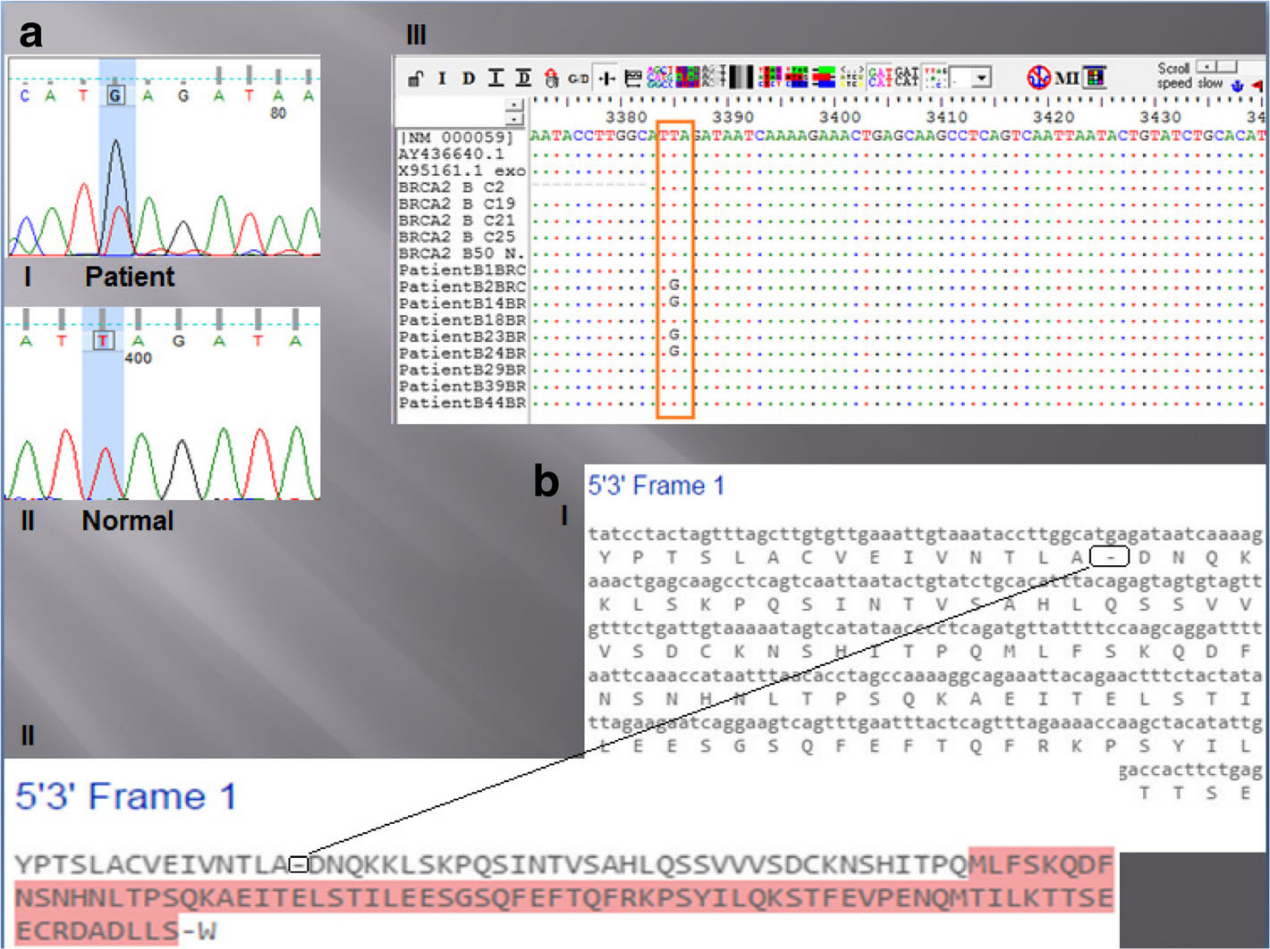
**a**: I. patient. Illustrates the sequencing result of the chromatogram of one of the tested patients with the substitution mutation marked by a small square. The monoallelic change is more apparent. II. Control. Illustrates the sequencing result of the chromatogram of the control with the normal sequence. A - Adenine, G - guanine, T - thymine, C - cytosine. III. Illustrates Bioedit multiple sequences alignment with substitution of thymine by guanine. **b**: I. This frame illustrates the nucleotide sequence (in small letters) and their corresponding amino-acids sequence (in capital letters) of a selected frame (5'﻿ to 3' frame 1) of the tested region (region B) of *BRCA2*. The dash (−) represents absence of amino-acid (stop codon). This figure was taken from Expasy online translate tool. II. this frame illustrates the amino-acids sequence in a compacted form. The dash (−) represents absence of amino acid. This figure was taken from Expasy online translate tool

By using the multiple sequence alignment tool BioEdit, the analysis of nine tested patients and five controls of the modified sequencing results -compared to NCBI RefSeq transcript mRNA (NM_000059.3) - revealed a single nucleotide change (substitution) within region B at position 3385 yielding a stop codon (TGA) in four patients as (TTA/TGA). The corresponding amino acid sequences appeared as gaps in (Fig. 2); in which the normal amino acid Leucine no longer existed as a result of premature termination (L1053X).

Another two single nucleotide changes had been noticed. The first one occurred in two patients with the previously noted L1053X and resulted in Adenine being replaced by Guanine at position 3474 (haplotype), and the corresponding amino acid change was N1083D. This variant was predicted to alter normal protein features in both function and structure -as shown by SIFT sequence and Project Hope. Also it was predicted to decrease protein stability -by I-Mutant. However, it was expected to probably harmless by MutationTaster2 and benign by polyphen-2. The other detected mutation -rs1801406- was silent (K1132 K) and noted in six cases, two of them had both L1053X and N1083D changes, (Table 4).

**Table 4:** Detected patients among the refined group to carry the following variants within *BRCA2* exon 11 primer B region

Patient ID	Age	Parturition	Origin	Tribe	Variants		
					Nonsense	Misense	Silent
					T3385G	A3474G	A3623G
B1	51	4	Central-kh^a^	Ja’alya			
B2	45	4	Western	Noba	Detected		Detected
B14	35	2	Northern^b^	Ja’alya	Detected	Detected	Detected
B18	41	5	Central-Kh	Kawahla			
B23	27	3	Central-G^c^	Ja’alya	Detected	Detected	Detected
B24	39	3	Western		Detected		
B29	37	2	Central-Kh	Bataheen			Detected
B39	30	5	Western	Kenany			Detected
B44	57	6	Western	Mema			Detected

^a^Khartoum

^b^River-Nile

^c^AlGezirah

### Nonsense mutations

**Fig. 3:**
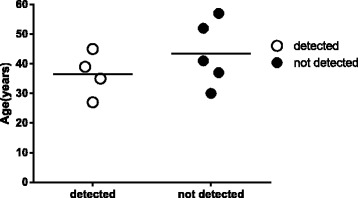
The mean age in breast cancer patients with and without the detected nonsense mutation

Patients carrying this mutation were premenopausal, with a mean parity of 3.0. The mean age of patients with and without the nonsense mutation was 36.5 and 40.5 years respectively, with a mean difference of four years as illustrated in (Fig. 3). Two patients bearing this SNP were from Ja’alya tribe and one of them had a history of secondary liver deposits (Table 3).

## Discussion

The significant change noted in this study was a monoallelic T3385G stop codon. A variant found with different nomenclatures, c.3158 T > G and n.3386 T > G (Table 5). This SNP was previously identified by Lubiniski in a study aimed to screen familial cases presented with seven different phenotypes including BC and Ovarian Cancer. He studied Ovarian Cancer Cluster Region (OCCR) within the *BRCA2* coding sequence. This region was noted more consistently to determine hereditary familial cancer cases. He found termination sequence at position T338**6**G [18, 36, 37]. The change was similar in both studies (T converted to G) but appears in different positions. However, the resulted-corresponding amino acid sequence provided the same change in both studies (L1053X). Also the mutation has been found as a germline-type but in prostatic cancer cases [38, 39] and one study found this variant within a control subject [40]. The geographic distribution of the variant within detected population has been covered (Table 6).

**Table 5:** highlights the stop codon L1053X with different nomenclatures described by ClinVar NCBI database

The study stop codon	SNP ID	Human Genome Variation Society HGVS	Breast Cancer Information Core BIC		References
			Nucleotide Accessions	Stop codon position	
T3385G, L1053X	rs41293477	c.3158 T > G	U43746.1	n.3386 T > G	[18] [40] [53] [39] [38]
			(RefeSeq) NM_000059.3	c.3385 T > G

**Table 6:** The geographic provenience of the samples previously detected with the mutation L1053X

SAMPLE geographic provenience	L1053X mutation frequency	Type	Cases	Age	The study highlighted the mutation	Sample source
Canada, USA and Poland^*1^	1 family (not specified)	Germline	Familial BC	-	Lubinski, et al. 2004 [18]	Research centers
UK, USA^*2^	1 control subject (not specified)		-	54	Song H, et al. 2014 [40]	Gayther SA, et al. 2007 [54]
Australia	1 case; as HRM High Resolution Melting Method validation	Method validation	Not specified	-	Hondow HL, et al. 2011 [53]	Peter MacCallum Cancer Centre and the Kathleen Cunningham Foundation Consortium for Research into Familial Breast Cancer (kConFab)
UK, Netherlands^*3^	1 case	Germline	Prostate ca	54.6	Sandhu SK, et al. 2013 [39]	Fong PC, et al. 2009 [55]
UK	1 case	Germline	Prostate ca with family history of BC and Lung ca	46	Kote-Jarai Z, et al. 2011 [38]	Eeles RA, et al.1997 [56]

^*1^Cancer centres where the sampling protocols including family pedigree were performed

^*2^based on large population studies: the population-based SEARCH study UK and the hospital-based Mayo clinic study from USA

^*3^The centers where the study was performed: at the Royal Marsden National Health Service (NHS) Foundation Trust (United Kingdom) and the Netherlands Cancer Institute (the Netherlands)

The patients carrying the mutation had a mean age of 36 years; similar to what was previously reported in Sudan by Awadelkarim et al. who analyzed 35 patients with breast cancer. In terms of parity and menopausal status of the subjects, both studies showed the same trend as the majority of BC cases were premenopausal and multiparous. Furthermore, patients from Ja’alya tribe were found to have truncating mutations in both studies [16].

Our mutation is located within the central region, which possesses eight functional BRC repeats to bind *RAD51* -that is essential for Homologous Recombination (HR)- to facilitate its loading onto single strand DNA, where a repair process is needed [41–44]. Accordingly, any defect of this loading will result in failure of Homologous recombination and the DNA double strand breaks remain altered [45].

From the NCBI database; *BRCA2* human has a total of about 10,736 known SNPs, and more than 466 reported truncating mutations. One of these mutations is the K3326X (rs11571833). This mutation has been associated with a 26% increase in the risk of developing breast cancer in European, Latin Americans, and Indian populations. K3326X mutation has been associated with a 2.5 fold increase in risk of squamous lung cancer [46]. Another example of stop codon mutation in *BRCA2* is Y3308X (rs4987049) which has been found in Asian, European, Sub-Saharan and African American populations. Other stop mutations in *BRCA2* coding region lack frequency data [47]. Seventy Nigerian breast cancer patients with ages younger than 40 years were studied, and one *BRCA2* truncating mutation 3034del4 within exon 11 has been reported [48]. The same mutation has been reported in a study of 39 early onset breast cancer (< 40 years) patients in Nigeria. Although 30 variants of *BRCA2* were detected, there was only one (3034del4) truncating mutation, located in exon 11 [49].

The N1083D mutation was not previously reported and such a companion is shown in this study by this variant regarding the position to be in continuation -sitting- few steps later after the monoallelic nonsense variant L1053X, so this position proves to be of no significance because it is situated after the nonsense mutation. The other variant, A3623G, was silently expressed as K1132 K, was detected with high frequency among earlier cases, and was involved with three cases detected with the nonsense L1053X including the two N1083D variants. The silent mutation K1132 K was reported among familial cases as the benign non-virulent bearing-characteristic and was found frequently within early onset <50 with mean age 37.5 and more frequently among Asian population and was noticed its high occurrence among a Chinese population [50, 51]. This variant has been recorded with other 13 variants as a recurrent situation among a Belgian population [52].

A technical facility to establish the outcome/resulting truncation inactivation is not available and it is very difficult to handle such a technical assessment. Though all 45 patients’ DNA had been extracted, only 10 patient’s extracts were sequenced owing to financial constraints. Also, due to these financial constraints only the product of one primer with the highest stability was subjected to further analysis in this study. Moreover, the sample size limits the generalizability of this study, but for this variant to be generalized to the Sudanese population, further studies using larger sample size will be needed in the future. In a general context, *BRCA* genes have not got wide assessment within our geographic region, thus in such scarce way of expression of BC genetic characteristics regarding some countries including Sudan, data presented in our study could be more raised. Most of *BRCA2* mutations variants detected within African literature have been gathered in (Table 7) with their corresponding country of origin.

**Table 7:** Most of the BRCA2 mutations variants detected within African literature

BRCA2 variants		Country	Ref.
c. 2826_2829delAATT		South Africa	van der Merwe NC, et al. 2012 [57]
c. 5771_5774delTTCA			
c. 6448dupTA			
c. 7934delG	Founder-		
c.5946delT			
C.8162delG			Schoeman M, et al. 2013 [58]
c.5999del4			
c.6174delT			
c.582G > A			Francies FZ, et al. 2015 [59]
c.5771_5774delTTCA			
c.5213_5216delCTTA			
c.8754 + 1G > A			
c.9097_9098insA			
c.4798_4800delAAT			
c.7712A > G			
c.9875C > T			
c.7934delG founder	founder	Afrikaner population of South Africa	Seymour HJ, et al.2016 [60]
c.6621delA		South Africa	
c.6761_6762delTT			
c.5073dupA		Morocco	LAARABI FZ, et al.2011 [61]
c.3381delT			Tazzite A, et al. 2012 [62]
c.7110delA			
c.7235insG			
c.517-1G > A			
c.6428 C > A			Guaoua S, et al.2014 [63]
c.745-1G > A			Jouhadi H, et al.2016 [64]
c.5682insA		Tunisia	Troudi W, et al. 2007 [65]
c.1309del4			
c.-25G > A			
c.6301 A > C			
c.1595 A > T			
c.7242 A > G			
c.865 A > G			
c.1310_1313del (1538delAAGA)			Fourati A, et al. 2014 [66]
c.-26G > A			Riahi A, et al.2014 [67]
c.681 + 56C > T			
c.793 + 65_793 + 65delT			
C.8503 T > C			
5456delGTAGCA			Hadiji-Abbes N,et al. 2015 [68]
c.1313dupT			Riahi A, et al.2015 [69]
c.7654dupT			
c.67 + 62 T > G			
c.8487 + 47C > T			
c.8360G > A			
c.8830A > T			
c.9875C > T			
c.10240A > G			
c.8182G > A			
c.8503 T > C			
c.1542_1547delAAGA			Riahi A, et al. 2017 [70]
c.5682insA			
c.1309del4			
c.1310 1313delAAGA		Algeria	Cherbal F, et al. 2010 [71]
c.5722 5723delCT			
c.67 + 14 T > C			
c.67 + 15 T > C			
c.68–14 T > A			
c.68-21 T > G			
c.231 T > G			
c.3555A > T			
c.3868 T > A			
c.5553C > T			
c.5472 T > G			
c.5592C > A			
c.5976A > G			
c.5985C > A			
c.8487 + 19A > C			
c.68-16 T > A			Cherbal F, et al.2012 [72]
c.475 + 25A > G			
c.794-5A > T			
c.1099G > A			
c.2636C > A			
c.2657A > G			
c.2673C > G			
c.5397A > T			
c.5428G > T			
c.6309A > C			
c.6346C > G			
c.9256G > A			
c.7654dupA			Henouda S, et al.2016 [73]
c.1528G > T			
Del exons 19–20			
c.6450del			
c.7462A > G			
c.1504A > C			
c.5939C > T			
c.1627C > A			
c.3195_3198delTAAT		Sudan	Awadelkarim KD, et al.2007 [16]
c.6406_6407delTT			
c.8642_8643insTTTT			
c.122C > T			
c.6101G > A			
c.68-7delT			
999TCAAA deleted (999del5)		Egypt	Bensam M, et al.2014 [74]
2256 T > C			
8934G > A			
c.970G > A		Nigeria	Fackenthal JD, et al.2005 [49]
c.1093A > C			
c.1503A > G			
c.2366 A > T			
c.3014 T > C			
c. 3188A > T			
c. 3199A > G			
c. 3492 T > C			
c. 4299A > C			
c. 4469C > T			
c. 4791G > A			
c. 5646A > G			
c. 5932G > A			
c. 5938C > G			
c. 6741C > G			
c. 7378C > A			
c. 7470A > G			
c. 7547A > G			
c. 9058A > T			
c. 9862G > C			
3034delACAA			
ex2-11C > T			
ex7-19C > T			
ex11-43 T > C			
ex12-200insC			
ex17-40A > G			
ex18 + 109G > A			
ex21-36C > G			
ex22-70C > T			
ex26 + 106delT			
1538delAAGA c.1310_1313delAAGA			Zhang J, et al.2012 [75]
1222delA			Fackenthal JD, et al.2012 [76]
2630del11			
3036delACAA			
4157delC			
5358delTGTA			
5369delATTT			
5469insTA			
5581delAC			
7482delAG			
9045delGAAA			
Q3066X			

## Conclusion

This study detected monoallelic L1053X mutation causing the same stop codon in *BRCA2* protein sequence at the same position in four Sudanese female BC patients out of nine from different families. This nonsense mutation should be evaluated in further studies in a larger number of BC patients in both hetero-homozygosity re-evaluation and to check the reliability of using this stop codon as a screening tool for early detection of BC.

## Abbreviations

BC: Breast Cancer
BLAST: Basic Local Alignment Search Tool
BRCA2: Breast Cancer type2
D: Aspartic acid
DNA: Deoxyribonucleic Acid
EDTA: Ethylenediaminetetraacetic acid
G: Guanine
HR: Homologous Recombination
K: Lysine
L: Leucine
mRNA: Messenger Ribonucleic Acid
N: Asparagine
NCBI: National Center for Biotechnology Information
OCCR: Ovarian Cancer Cluster Region
PCR: Polymerase Chain Reaction
RefSeq: Reference Sequence
RICK: Radiation and Isotope Center in Khartoum
SIFT: Sorting Intolerant from Tolerant
SNP: Single nucleotide polymorphism
T: Thymine
TE buffer: Tris-EDTA
X: Termination stop codon
Y: Tyrosine
